# Mechanism of Abnormal Coagulation Induced by Tigecycline in Cancer Patients

**DOI:** 10.3389/fphar.2022.891952

**Published:** 2022-07-05

**Authors:** Li-Hua Sun, Kun-Hao Bai, Guo-Yan Wu, Xiao-Peng Tian, Zhi-Qing Zou, Da-Wei Wang, Yu-Jun Dai, Si-Liang Chen

**Affiliations:** ^1^ Department of Hematology, Peking University Shenzhen Hospital, Shenzhen, China; ^2^ State Key Laboratory of Oncology in South China, Collaborative Innovation Center for Cancer Medicine, Guangzhou, China; ^3^ Department of Endoscopy, Sun Yat-Sen University Cancer Center, Guangzhou, China; ^4^ Department of Critical Care Medicine, Shanghai General Hospital, Shanghai Jiao Tong University School of Medicine, Shanghai, China; ^5^ Jiangsu Institute of Hematology, The First Affiliated Hospital of Soochow University, Suzhou, China; ^6^ State Key Laboratory of Medical Genomics, Shanghai Institute of Hematology, National Research Center for Translational Medicine at Shanghai, Ruijin Hospital Affiliated to Shanghai Jiao Tong University School of Medicine, Shanghai, China; ^7^ Department of Hematologic Oncology, Sun Yat-Sen University Cancer Center, Guangzhou, China

**Keywords:** tigecycline, abnormal coagulation, cancer, RNA-Seq, CHO

## Abstract

Tigecycline is a broad-spectrum active intravenous antibiotic that is active against methicillin-resistant staphylococcus aureus. In Phase 3 and 4 clinical trials, increased all-cause mortality was observed in patients treated with tigecycline compared to patients in the control group. The reason for the increase is unclear. In this study, we found that tigecycline cause abnormal coagulation in tumor patients, especially in patients with hematological malignancies. The main manifestations were decreased fibrinogen and prolonged activated prothrombin time (APTT), thrombin time (TT), and D-dimer. In addition, through functional studies, we found that tigecycline inhibit platelet adhesion and aggregation, and the coagulation function of patients gradually recover after discontinuation. Gene sequencing results suggested that tigecycline significantly regulate the expression of genes related to platelet function pathways and increase the incidence of single nucleotide polymorphisms and the number of alternative splices in the Chinese hamster ovary (CHO) cells treated with tigecycline. An abnormal function and low numbers of platelets are common in patients with hematological malignancies. Our study can explain the mechanism of abnormal coagulation caused by tigecycline. Additionally, doctors who apply tigecycline to cure infections in tumor patients should be warned.

## Introduction

Patients with hematological malignancies often suffer from dysfunctions of coagulation and fibrinolysis systems, and coagulation dysfunctions are a common pathophysiological change (Röllig and Ehninger, 2015). In normal organisms, coagulation and anticoagulation are in a dynamic balance, which can be broken by enhanced coagulation activity or weakened anticoagulation activity. In patients with acute leukemia (AL), due to the consumption of coagulation factors or the existence of coagulation factor inhibitors, the content or activity of some coagulation factors is reduced, resulting in disorders of coagulation systems; pathological bleeding has become one of the most common clinical symptoms (Just Vinholt et al., 2019; Karlström et al., 2022). Current studies have also suggested that coagulation dysfunctions are one of the important causes of early death in AL patients (Libourel et al., 2016; Chatzidimitriou et al., 2021). Especially in acute promyelocytic leukemia (APL), bleeding is the leading cause of early death in patients (Testa et al., 1978). In addition, thrombosis is common in AL patients. Some patients have both bleeding and thrombosis, reflecting the complexity of the pathogenesis of coagulation disorders.

As a common complication of AL after chemotherapy, infection secondary to sepsis is one of the significant causes of death of AL patients after chemotherapy (Halim et al., 2007; Röllig and Ehninger, 2015). Infectious sepsis is closely related to neutropenia caused by AL, mucosal damage by chemotherapy drugs, irregular use of antibiotics, and long hospitalization cycles (Tramsen et al., 2016). Sepsis caused by infection can interfere with coagulation systems (Bochennek et al., 2016). With the aggravation of infection, the correlation between immune-inflammatory response and coagulation cascade is further strengthened; infection-related indexes and coagulation system activation synergistically worsen the condition of patients with sepsis (Halim et al., 2007).

Tigecycline is the first glycyl-tetracycline antibiotic used in the clinic. It binds to the A sites of the 30S subunits of bacterial ribosomes, blocking bacterial transcription to inhibit protein synthesis (Senior, 2005). Tigecycline shows a strong advantage in treating drug-resistant bacteria because of its excellent antibacterial activity. However, it has many adverse effects, mainly in the form of anaphylaxis/anaphylactoid reactions, acute pancreatitis, liver cholestasis, and jaundice (Cai and Wang, 2011). In controlled clinical studies, the incidences of both infection-related serious adverse events and sepsis/septic shock were higher in the tigecycline-treated group than in the control group (Bucaneve et al., 2014; Montravers et al., 2014). Due to baseline differences in this subgroup of patients, the relationship between outcomes and treatment is not yet clear.

Recent studies demonstrated that tigecycline induce coagulopathy in severely infected patients, especially those with hepatic or renal impairment (Cui et al., 2019; Leng et al., 2019; Guo et al., 2022). However, adverse reactions of tigecycline after use in cancer patients are rarely reported. In this study, we found that using tigecycline in tumor patients cause changes in coagulation indexes. An abnormal coagulation function could aggravate the condition of patients, prolong hospitalization, and cause a poor prognosis. In addition, we further explored the mechanism of abnormal coagulation induced by tigecycline to provide safe anti-infective treatment for tumor patients.

## Materials and Methods

### Patient Sample Data

With the help of the hospital information system, we screened the cases of hospitalized tumor patients using tigecycline in Sun Yat-sen University Cancer Center from November 2015 to October 2019 using a retrospective analysis method. The exclusion criteria: 1) patients with hospitalization periods <4 days; 2) patients with severe hepatic insufficiency (Child-Pugh class C); 3) pregnant or lactating women; 4) patients with abnormal coagulation or congenital coagulation disorders such as hemophilia before hospitalization; 5) patients using other drugs that affect coagulation indexes 2 weeks before or during the medication; 6) patients without the detection indexes of coagulation function before and after the medication; 7) patients with incomplete medical records. All participants provided written informed consent according to the regulations of the Institutional Review Boards of the Hospitals in agreement with the Declaration of Helsinki with the number SL-B2021-340-01.

### Observation Indexes

The main safety observation indexes: the coagulation function-related indexes during hospitalization, including prothrombin time (PT) before and after treatment, prothrombin time activity (PT%), international standardization ratio (INR), partially activated prothrombin time (APTT), fibrinogen (FBG), thrombin time (TT), D-dime and fibrin degradation product (FDP). Minor safety observation indexes: the levels of alanine aminotransferase (ALT), aspartate aminotransferase (AST), creatinine (CRE), and C-reactive protein (CRP) before and during the treatment with tigecycline.

We defined the abnormal coagulation index as the APTT higher than the normal value of 10 s (i.e., APTT >50 s), the PT and TT higher than the normal values for 3 s (i.e., PT > 17 s and TT > 24 s), or the FBG lower than the normal value (i.e., FBG < 2 g/L) and paid attention to the platelet count (PLT) lower than normal value (PLT <100×10^9^/L). In logistic regression analysis, patients with the ages of < years old, 19–60 years old, and ≥61 years old were divided into the adolescent group, the normal age group, and the elderly group, respectively; patients with the tigecycline use times of 7 days, 8–14 days, and >14 days were divided into the medium-course treatment group, the medium-long-course treatment group, and the long-course treatment group, respectively; patients with both ALT and AST ≤50 IU/L were considered to have a normal liver function, and patients with ALT or AST >50 IU/L were considered to have an abnormal liver function; patients with CRE ≤106 μmol/L were considered to have a normal renal function, patients with CRE >107 μmol/L were considered to have an abnormal renal function.

### Cell Adhesion Assay

The cell *adhesion* assay was performed as previous described (Huang et al., 2015). Chinese hamster ovary cells were obtained from Da-Wei Wang (Ruijin hospital) and cultured in F12 (Gibco, NY, United States) supplemented with 10% fetal bovine serum (Gibco, 10270-106) and 2 mM Glutamine (Gibco, NY, United States). The cells were seeded into 96-well plates at a density of 10^5^ cells/well. To determine the biological effect of the tigecycline (Hansoh Pharma, Jiangsu, China), cells were separately incubated with appropriate concentrations (0 mg/ml, 0.2 mg/ml, and 0.5 mg/ml) of tigecycline. After 48 h, 10 μL of reagent from a Cell Counting Kit-8 (CCK-8, Dojindo Laboratories, Kumamoto, Japan) was added to each well. The samples were then incubated at 37°C for an additional 4–6 h and measured at the absorbance of 450 nm using a spectrophotometer.

### Cell Spreading Assay

The cell spreading assay was performed as previous described (Huang et al., 2015). The tigecycline was diluted into three groups of 0 mg/ml, 0.2 mg/ml, and 0.5 mg/ml and treated in CHO cells for 48 h. Then, 10 mg/ml BSA was added to a 96-well plate at 50 μL/well, coated at 4 °C overnight, washed twice with DPBS the next day, and blocked with 20 mg/ml BSA at room temperature for 2 h. The CHO cells treated with tigecycline for 48 h were digested and made into a single-cell suspension, washed twice with 1 ml of F12 medium, and then washed with 1 × 10^6^/ml density suspended in the medium. Next, 100 μL of cell suspension was added to the coated 96-well plate. Each group of cells was connected to 3 replicate wells, placed in a 37°C, 5% CO_2_ cell incubator for 1–2 h, and then observed under the microscope when the CHO cells were well stretched. Incubation was terminated, and non-adherent cells were removed by being washed three times with DPBS. After being washed with DPBS, pictures were taken under a microscope, and ImageJ software was used to calculate the area of single cells one by one. We defined cells with an area of ≥30 μm^2^ as stretching, and cells with an area of <30 μm^2^ as adherent only. The total number of cells in each group was about 300, and the percentage of cells of each shape in all groups were calculated. Fluorescence microscopy was used to reflect cell viability, and we used cell staining to make cells appear green under fluorescence, while dead cells were not.

### RNA-Sequencing and Pathway Analysis

The CHO cells treated with tigecycline (0 mg/ml and 0.2 mg/ml) for 48 h were used for RNA-sequencing. KOBAS (http://kobas.cbi.pku.edu.cn/home.do) was used for the KEGG pathway enrichment analysis, in which the calculation principle is the same as that in GO functional enrichment analysis, and the calculation is performed using Fisher’s exact test (Wu et al., 2006). In order to control the calculated false positive rate, the BH (FDR) method was used for multiple testing, and the calculation formula was not the same as that in the previous section. The corrected P-value threshold was 0.05.

### Statistical Analysis

SPSS 22.0 statistical software was used for analysis. Measured data were expressed as mean ± standard deviation and were tested for homogeneity of variance. When the variance was homogeneous, a *t*-test was used, and when the variance was unequal, a corrected *t*-test was used. A rank-sum test or Ridit test was used for grade data. The categorical data were analyzed using the χ2 test. According to α = 0.05 level, *p* < 0.05 implied a statistical difference, and *p* > 0.05 implied no statistical difference.

### Thromboelastography

The function of platelet was detected by thromboelastography as previous studies (Wang et al., 2014; Guo et al., 2021; Miles et al., 2022; Sekar et al., 2022). Assays were performed according to the manufacturer’s instructions (Hemostasis System Kaolin Testing Kit, Glodmag, China). Briefly, samples were fresh whole blood, 2 ml was collected with citric acid anticoagulation, and kept at room temperature for at least 15 min. Then, draw 340 ul blood sample for on-machine testing (Thrombelastography TEG5000, HAS). All samples were detected and analyzed within 2 h after sampling. The main indicators of thromboelastometry were: (1) Reaction time (R) indicated that there was no fibrin formation in the tested sample; (2) Coagulation time (K) indicated that fibrin began to form in the tested sample, which had a certain firmness; (3) The widest distance (MA) on both sides of the curve represented the maximum amplitude of thrombus formation; (4) Thromboelastometry (ε), which represented the size of the elasticity of the thrombus. (5) Maximum coagulation time (m) indicated the time from coagulation time to the maximum amplitude.

## Result

### The Effect of Tigecycline on Coagulation in Tumor Patients

**FIGURE 1:**
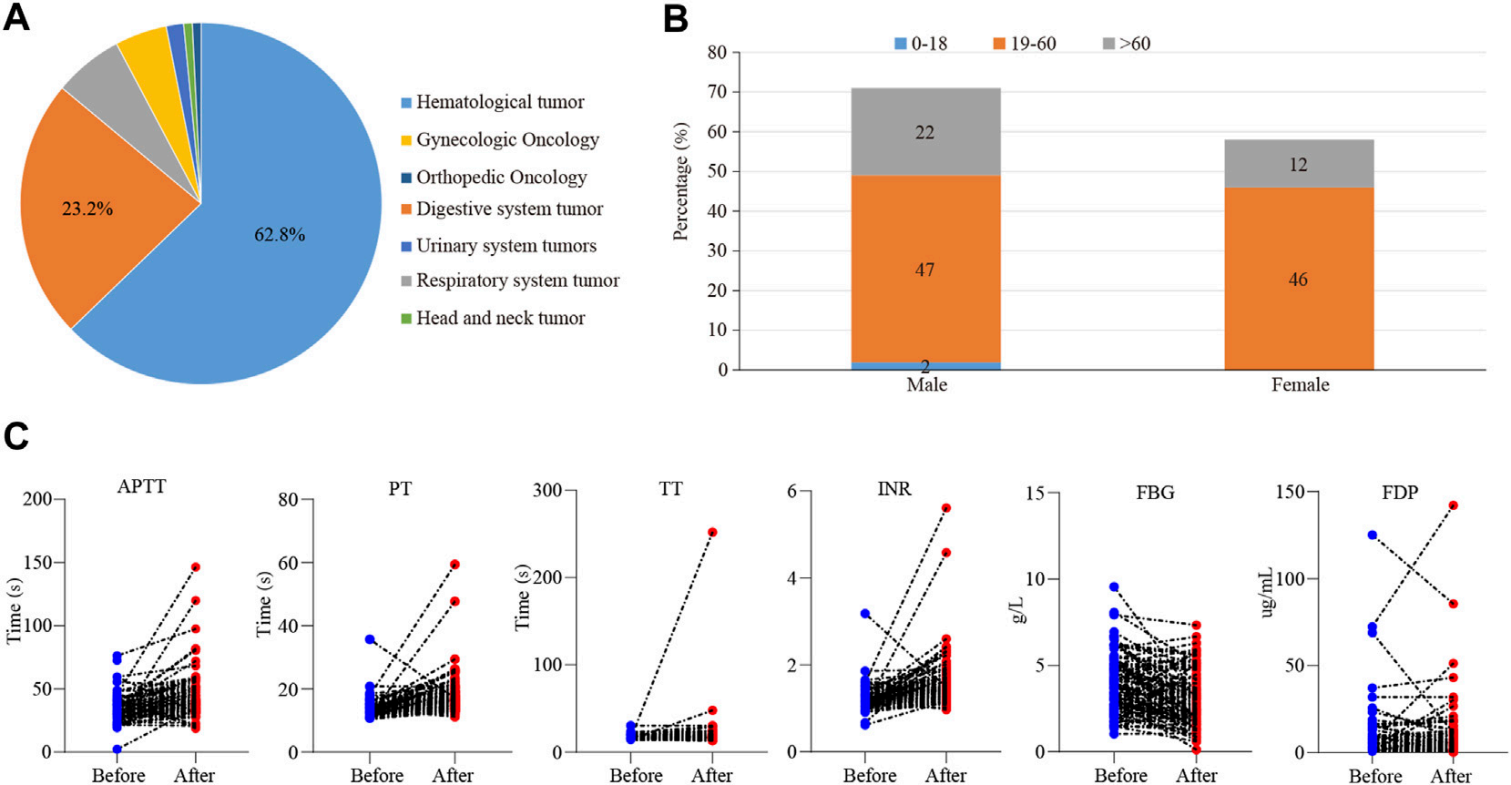
The effect of tigecycline on coagulation in tumor patients. **(A)** The pie analysis of types of cancers involved in this retrospective analysis. **(B)** The statistical histogram of gender and age of the patients treated with tigecycline. **(C)** The blood coagulation indexes of hematological tumor patients before and after tigecycline treatment.

A total of 205 cases of tigecycline used in tumor patients were recruited from our hospital, and 129 valid cases were finally included. Basic characteristics of this cases were shown in Figure 1. Among them, the most common patients were hematological tumors, accounting for about 62.8% (81 cases), followed by digestive system tumors, accounting for about 23.2% (Figure 1A). There were 71 males (55%) and 58 females (45%). The average age was (49.5 ± 15.9) years. There were 2 cases (1.5%) in the adolescent group (age <18 years), 47 cases (72.1%) in the normal age group (19–60 age), and 22 cases (26.4%) in the elderly group (≥61 years old) (Figure 1B). In addition, the days of tigecycline administration were 10.58 ± 9.12days and we found 113 cases (87.6%) had abnormal coagulation function.

Then, we further examined which coagulation indexes were affected by tigecycline. After medication, the coagulation indexes, mainly including PT, INR, APTT, TT, D-Dimer, and FDG, showed an upward trend (Figure 1C). As shown in Table 1, the coagulation function of the tumor patients before using tigecycline was near the normal baseline. However, after 3 days of tigecycline administration, the PT of the patients was significantly prolonged (17.56 ± 5.72 s) and aggravated with the prolongation of the administration time. After continuous administration for more than 14 days, the PT of the tumor patients was abnormally prolonged (19.91 ± 12.75 s), and even the lives of the patients might be threatened. The FBG of the patients before tigecycline treatment was 3.98 ± 3.45 g/L and showed a downward trend at different time points after treatment. The FBG decreased significantly after 1–3 days, 4–7 days, 8–14 days, and more than 14 days of medication (*p* < 0.05). The PT% and FBG decreased in cancer patients with tigecycline treatment, while the TT, APTT, and D-Dimer significantly increased after treatment, and this effect did not increase with the prolongation of the administration time. In addition, the INR also increased after treatment. However, unlike others, the INR peaked within 3 days after administration, then decreased slowly, and remained stable at a relatively high level. Further, the FDP peaked on d 4–14 of tigecycline administration and then gradually declined (Table 1).

**TABLE 1:** The coagulation parameters in peripheral blood of cancer patients with tigecycline treatment.

Time	n	PT (s)	PT%	INR	APTT (s)	Fbg (g/L)	TT (s)	D-Dimer (ug/mL)	FDP (ug/mL)
Before	129	13.50 ± 2.65	78.26 ± 20.56	1.21 ± 0.25	33.67 ± 9.75	3.98 ± 3.45	17.25 ± 2.41	8.94 ± 1.54	25.61 ± 9.45
Day 1-3	129	17.56 ± 5.72	53.66 ± 16.81	1.56 ± 0.55	42.01 ± 16.29	3.07 ± 1.56	20.14 ± 2.33	9.21 ± 1.9	26.80 ± 9.49
Day 4-7	97	16.58 ± 4.74	57.45 ± 16.85	1.47 ± 0.46	38.40 ± 14.92	3.42 ± 1.87	19.41 ± 2.11	9.29 ± 2.86	29.99 ± 7.13
Day 8-14	57	16.54 ± 5.41	58.03 ± 18.48	1.44 ± 0.51	41.12 ± 13.56	3.08 ± 2.73	18.46 ± 3.41	9.70 ± 2.06	29.29 ± 7.78
Day>14	10	19.91 ± 12.75	56.22 ± 21.72	1.50 ± 0.53	41.32 ± 9.00	2.58 ± 1.40	20.20 ± 6.93	9.52 ± 1.12	25.81 ± 6.43

### The Effect of Tigecycline on the Function of Platelets

**FIGURE 2:**
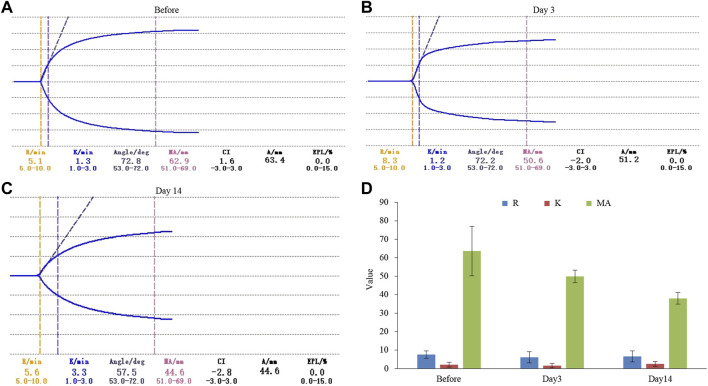
The thromboelastography analysis of hematological tumor patients with tigecycline treatment. **(A)** The result of thromboelastography of patients before using tigecycline. **(B)** The result of thromboelastography of patients after 3 days of treatment with tigecycline. **(C)** The result of thromboelastography of patients after 14 days of treatment with tigecycline. **(D)** The statistical analysis of thromboelastography in patients (*n* = 10). Reaction time (R) indicated that there was no fibrin formation in the tested sample; Coagulation time (K) indicated that fibrin began to form in the tested sample, which had a certain firmness; The widest distance (MA) on both sides of the curve represented the maximum amplitude of thrombus formation.

**FIGURE 3:**
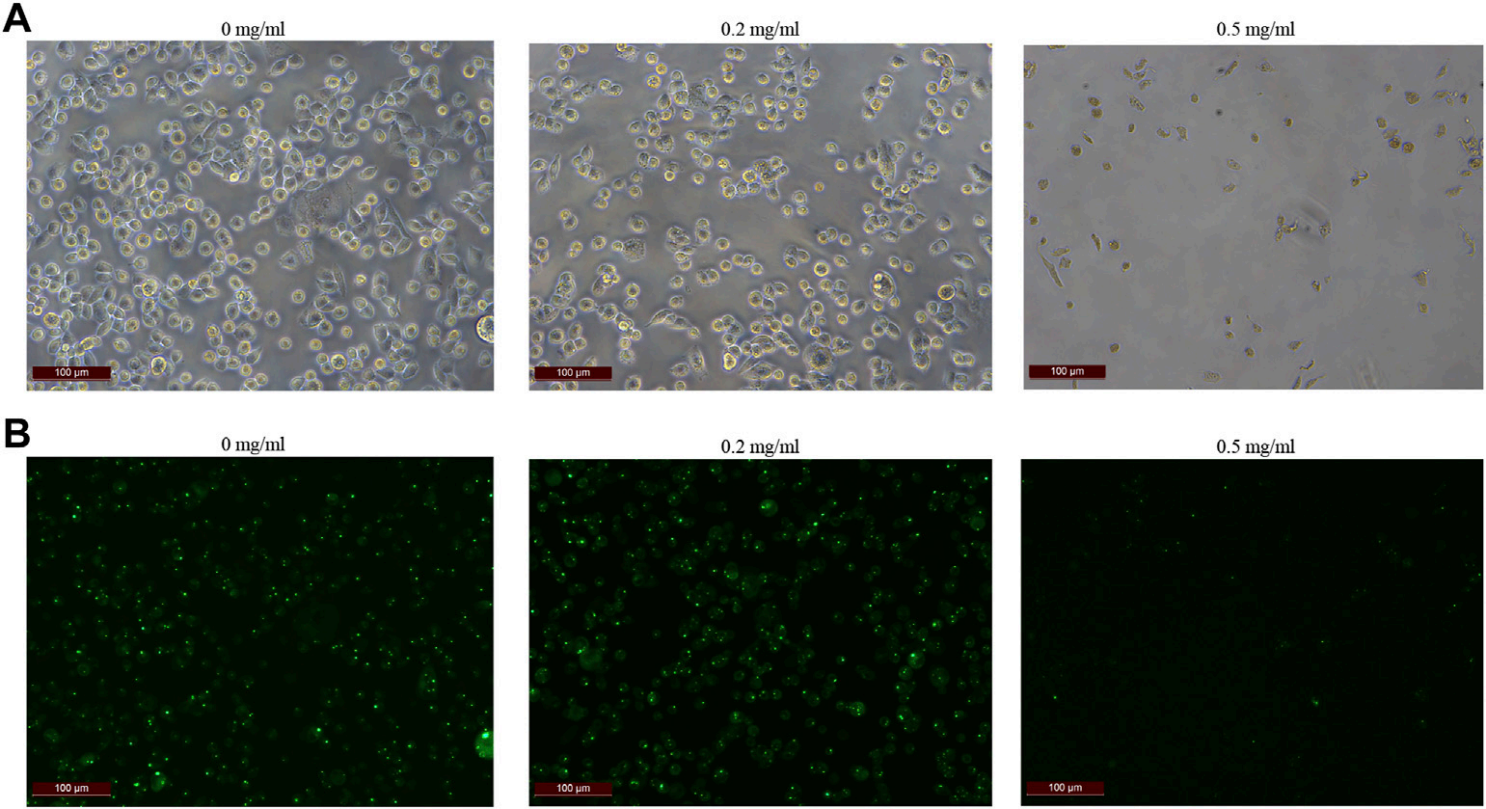
The cell extension of CHO cells *in vitro*. **(A)** The cell extension results of CHO cells treated with tigecycline with the concentrations of 0 mg/ml, 0.2 mg/ml, and 0.5 mg/ml. **(B)** Fluorescence microscopy was used to reflect cell viability, and we used cell staining to make cells appear green under fluorescence, while dead cells were not. The fluorescence color results of CHO cells treated with tigecycline with the concentrations of 0 mg/ml, 0.2 mg/ml, and 0.5 mg/ml.

In order to further explore how tigecycline causes abnormal coagulation function in tumor patients, we retrospectively analyzed the liver function and platelet function of these tumor patients according to our screening conditions. Our results showed that tigecycline did not affect the liver and kidney function of patients (Table 2). Both the ALT and AST tended to decrease after administration, which might be related to the infection status of patients before treatment. The ALT/AST ratios and serum creatinine did not change significantly before and after treatment (Table 2). We further detected and compared the changes of coagulation function indexes in tumor patients before and after the use of tigecycline by thromboelastography. The R value remained within the normal range (5-10) before and after the use of tigecycline with 5.1 (Before, before use of tigecycline), 8.3 (Day 3, 3 days after use of tigecycline), and 5.6 (Day 14, 14 days after use of tigecycline), respectively. However, the K value did not change significantly after using tigecycline for 3 days, but increased significantly after 14 days of using tigecycline. The prolongation of the K value indicated that the patient was in a hypocoagulable status. In addition, the MA value began to decrease after the third day of use of tigecycline, and further decreased with the prolongation of the medication time with 62.9 (Before), 50.6 (Day 3), and 44.6 (Day14), respectively. The decreased MA value reflected that tigecycline induce platelet function insufficiency (Figure 2). Next, we used CHO cells to explore the mechanism of tigecycline on coagulation function *in vitro*. We defined cells with an area of ≥30 μm^2^ as stretching, and cells with an area of <30 μm^2^ as adherent only. The results of the cell spreading experiment showed that the average numbers of CHO cells in the three groups of tigecycline with different concentrations (0 mg/ml, 0.2 mg/ml, and 0.5 mg/ml) were 226, 266, and 4, respectively. The percentages of cells with stretched morphology in the three groups were 73.14, 75.78, and 40%, respectively (Figure 3). In addition, the results of the cell adhesion experiment showed that the numbers of the adherent cells also decreased with the increase in the concentration of tigecycline. However, the percentages of cells with adherent morphology increased significantly, which were 26.86% (0 mg/ml), 24.22% (0.2 mg/ml), and 60% (0.5 mg/ml), respectively (Table 3).

**TABLE 2:** Liver and kidney function of cancer patients with tigecycline treatment.

Time	n	ALT (U/L)	AST (U/L)	AS/AL	Cre (μmol/L)
Before	129	70.24 ± 43.40	161.90 ± 13.01	1.45 ± 0.92	67.73 ± 9.82
After	72	48.29 ± 12.42	77.14 ± 21.53	1.60 ± 0.51	79.81 ± 10.57

**TABLE 3:** Cell adhesion experiments.

CHO(0 mg/ml)	CHO(0.2 mg/ml)	CHO(0.5 mg/ml)
0.7794	0.5136	0.0376

### The Genes Regulated by Tigecycline

**FIGURE 4:**
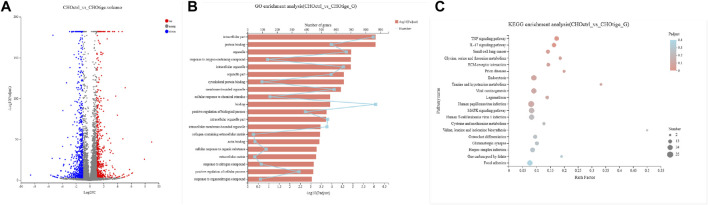
The gene functional analysis of tigecycline by RNA-sequencing. **(A)** The volcano plot analysis of differentially expressed genes. Each point in the figure represents a specific gene. Red points represent significantly up-regulated genes, blue points represent significantly down-regulated genes, and gray points represent non-significantly different genes. **(B)** The GO functional enrichment analysis of differentially expressed genes between CHO cells treated with and without tigecycline. **(C)** The KEGG enrichment analysis of differentially expressed genes between CHO cells treated with and without tigecycline.

To further study the mechanism of tigecycline regulating coagulation abnormalities, we harvested CHO cells treated with tigecycline with the concentrations of 0 mg/ml and 0.2 mg/ml for 48 h for RNA-sequencing. We used the software edgeR to analyze the differentially expressed genes. Based on the gene read-count data, the differential expression was calculated, and the significantly differentially expressed genes were screened (FDR <0.05 and |log2FC| ≥ 1) (Figure 4A). According to the criteria, we screened out a total of 1,269 genes that meet the requirements (Supplementary Table S1). The GO functional enrichment analysis of these differentially expressed genes indicated that membrane-bounded organelle, cellular response to chemical stimulus, and binding pathways were more enriched in the group with tigecycline treatment (Figure 4B and Supplementary Table S2). Then, KEGG pathway enrichment analysis was performed using KOBAS, and calculation was performed using Fisher’s exact test. We found that the expression of genes involved in the TNF signaling pathway, IL-17 signaling pathway, and Focal adhesion was significantly increased after tigecycline treatment (Figure 4C and Supplementary Table S3). Additionally, bulk RNA-sequencing assay showed significant abnormal expression in gene sets associated with platelet-activation and coagulation, such as *PKFP, PLA2g7, PDGFRA, PDGFRB, PAFAH1b3, PDGFA, PECAM1, PDGFC*, and *MPIG6b.* Furthermore, adhesion regulating molecules *RPN13, CHL1, ICAM5,* and *TLCN* were also down-regulated by tigecycline in CHO cells. These key genes shown above need to be further validated in blood samples of acute myelocytic leukemia patients treated with tigecycline.

### The Single Nucleotide Polymorphisms and Alternative Splicing Events Affected by Tigecycline

**FIGURE 5:**
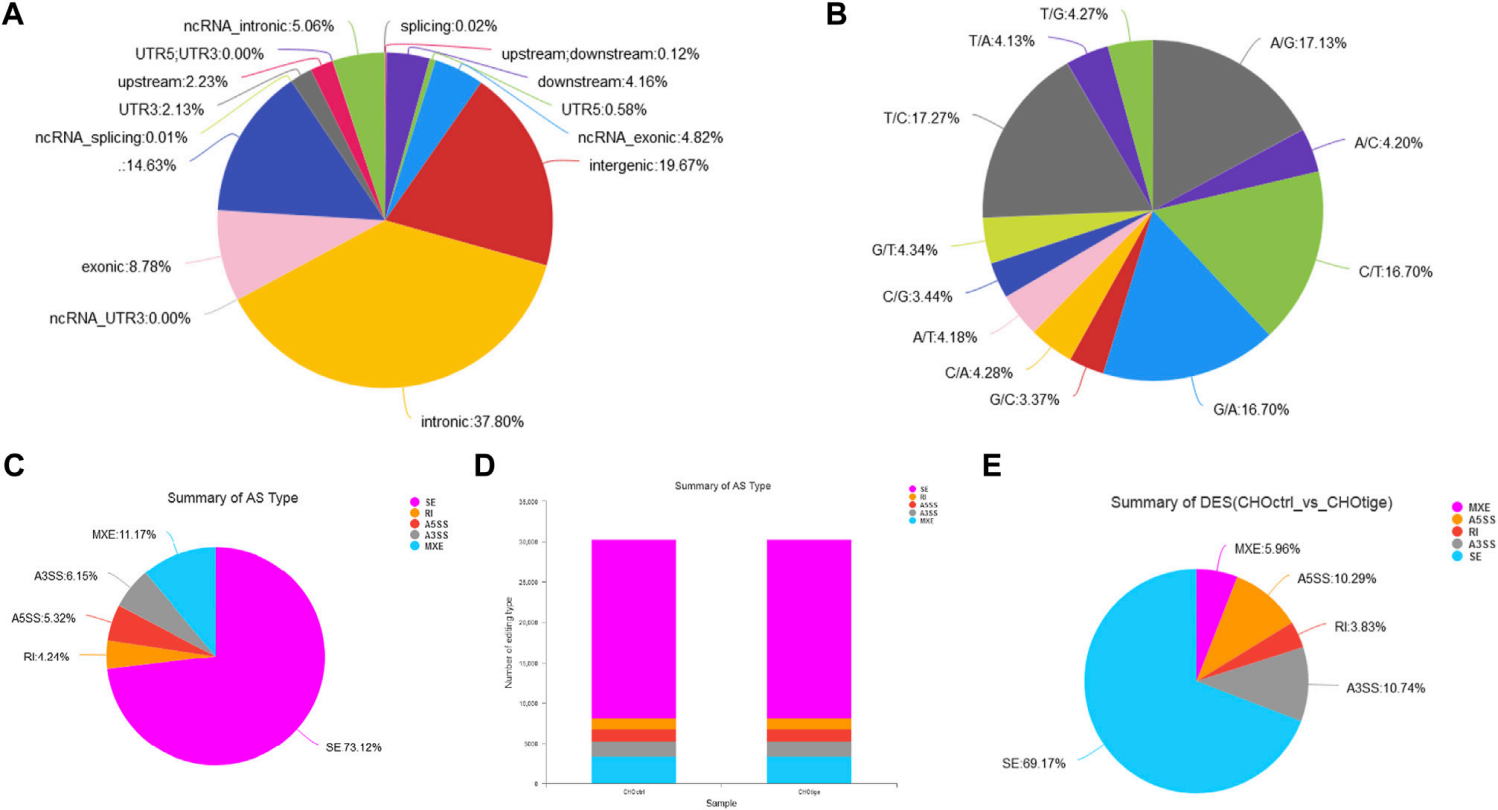
The analysis of SNPs and AS events of CHO cells with tigecycline treatment by RNA-sequencing. **(A)** The SNP distribution in genome regions. **(B)** The summary of SNP types in CHO cells with tigecycline treatment. **(C)** The statistics of AS events in one sample. **(D)** The statistics of AS events in two groups. **(E)** The statistical pie chart of types of AS events.

The SNPs and AS events can influence gene expression and the corresponding downstream functions (Zimdahl et al., 2004; Chen et al., 2020). Here, we further explored the SNPs and AS events in CHO cells with tigecycline treatment. We used the assembled transcript as a template sequence and aligned the template sequence with the original sequence using Samtools (http://samtools.sourceforge.net/) and VarScan v.2.2.7 (http://varscan.sourceforge.net/) software to find candidate SNPs. We found no significant differences in SNP types and distribution in CHO cells before and after treatment with tigecycline (Figures 5A,B, Table 4 and Supplementary Table S4). In addition, the splicing analysis indicated that the number of AS events was near 30,000, and skipped exon (SE) was the most common type in the two groups (Figures 5B,C). The pie chart of differentially expressed AS events in these two groups showed that SE (69.17%) was also the most occupied subtype, followed by alternative 3’splice site (A3SS,10.74%) and alternative 5’splice site (A5SS, 10.29%) (Figure 5C).

**TABLE 4:** SNP_position_distribution of CHO cells.

Region	CHOctrl	CHOtige
splicing	30	35
upstream;downstream	169	165
downstream	5664	5731
UTR5	788	792
ncRNA_exonic	6564	6510
intergenic	26792	27975
intronic	51491	50706
ncRNA_UTR3	2	2
exonic	11959	11938
.	19930	19763
ncRNA_splicing	8	8
UTR3	2905	2915
upstream	3039	2991
UTR5;UTR3	4	4
ncRNA_intronic	6889	6788
total	136234	136323
A/G	19922	20060
A/C	4879	4923
C/T	19415	19344
G/A	19419	19528
G/C	3916	3917
C/A	4973	4965
A/T	4864	4904
C/G	4003	3927
G/T	5043	5008
T/C	20084	20117
T/A	4802	4821
T/G	4967	5029
Total	116287	116543

## Discussion

Due to the broad-spectrum and effective antibacterial activity of tigecycline, it has been used to treat many patients with severe infections, and the range of indications has continued to expand (Alsaad et al., 2014). With the wide clinical applications of tigecycline, more and more reports of its adverse events have been reported. However, the reports of coagulation disorders are limited (Cai and Wang, 2011; Libourel et al., 2016; Brandtner et al., 2020). In the retrospective analysis of post-infection cancer patients treated with tigecycline, we found that patients treated with tigecycline had significant coagulation abnormalities that were irreversible by fresh plasma infusion. During the medication, the blood of patients might present a hypocoagulable state and continue to progress, resulting in life-threatening bleeding; after drug withdrawal, coagulation indexes quickly return to normal. Almost all patients with normal or slightly higher FIB levels before treatment had significantly decreased FIB levels after treatment; the PT, APTT, and TT were all prolonged compared with those in the control group; the PLT did not change significantly. The mechanism of their occurrences is still unclear. As we known, PAI-1 and tPA were important indicators for evaluating platelet function (Zheng et al., 2020). Unfortunately, we were currently unable to obtain relevant data from the recruited patient information.

Tigecycline competed with bacterial tRNAs to bind tightly to the 30S subunits of the bacterial ribosomes, blocking their connection with the bacterial ribosomes to affect bacterial protein synthesis and inhibit bacteria (Kumarasamy et al., 2010; Skrtic et al., 2011). The thromboelastometry results showed that tigecycline could directly cause hypocoagulation without no significant relationship with the number of platelets. Some reports showed that vitamin K supplementation effectively improve tigecycline-related coagulation dysfunctions. We wondered if tigecycline might affect the coagulation function of patients by affecting the synthesis of vitamin E and directly acting on the coagulation cascade (Provinciali et al., 2011). However, consistent with other studies, we observed that only a few cases showed elevations in transaminases and total bilirubin after medication, but no liver damage was clearly identified in tumor patients. Therefore, the resulting decrease in liver synthesis could not explain the hypocoagulant phenomenon. Although CHO cells were derived from hamster ovaries, their genomes were not exactly matched to those of humans. The feature that CHO cells could be cultured in suspension and adherent at the same time made them be one of the important cells for platelet function research, and had been widely used in platelet function research in the world (Grant et al., 2017; Ji et al., 2017). We used CHO cell lines to study the mechanism of gene expression and regulation patterns and found that tigecycline could regulate platelet activation and coagulation and adhesion regulating molecule-related genes.

For patients with hematological tumors, the hematopoietic system is abnormal, and platelet count and function are significantly lower than those of normal people. In addition, treatment with many chemotherapeutic agents such as tamoxifen, carboplatin or oxaliplatin, targeted therapy and radiotherapy also led to thrombocytopenia (Erman et al., 2004; Naveed Ahmad et al., 2022). Thus, it is better to avoid overdose and long-term treatment of tigecycline. For cancer patients with agranulocytosis complicated by infection after chemotherapy, targeted therapy or radiotherapy, various coagulation indicators (such as FIB, TT, APTT, INR and PLT, etc.) should be monitored when using tigecycline.

## Conclusion

In summary, we found serious adverse reactions related to procoagulant functions during using tigecycline in cancer patients. Tigecycline should be discontinued immediately once a coagulation dysfunction is found, and patients with severe disease and bleeding should be transfused with fresh frozen plasma, cryoprecipitate or FIB and given corresponding hemostatic therapy.

## Data Availability

The datasets presented in this study can be found in online repositories. The names of the repository/repositories and accession number(s) can be found below: https://www.ncbi.nlm.nih.gov/geo/, GSE198830.

## Supplementary Material

Supplementary Table S1 The coagulation indexes in peripheral blood of cancer patients with tigecycline treatment.

Supplementary Table S2 Liver and kidney function of cancer patients with tigecycline treatment.

Supplementary Table S3 Cell adhesion experiments.

Supplementary Table S4 SNP distribution of CHO cells.

## Glossary

APTT: activated prothrombin time
AL: acute leukemia
APL: acute promyelocytic leukemia
ALT: alanine aminotransferase
AST: aspartate aminotransferase
AS: alternative splicing
A3SS: alternative 3’splice site
A5SS: alternative 5’splice site
CRE: creatinine
CRP: C-reactive protein
CCK-8: cell counting kit-8
CHO: chinese hamster ovary
DES: differential expressed splicing
DPBS: dulbecco’s phosphate-buffered sallines
FBG: fibrinogen
FDP: fibrin degradation product
FDR: false discovery rate
FIB: fibrinogen
GO: gene ontology
INR: international standardization ratio
K: coagulation time
KEGG: kyoto encyclopedia of genes and genomes
KOBAS: KEGG orthology based annotation system
MA: the widest distance on both sides of the curve represented the maximum amplitude of thrombus formation
MXE: mutually exclusive exons
PT: prothrombin time
PT%: prothrombin time activity
PLT: platelet count
PAI-1: plasminogen activator inhibitor-1
R: reaction time
RI: retain intron
SNPs: the single nucleotide polymorphisms
SE: skipped exon
TT: thrombin time
TEG: thromboelastography
ε: thromboelastometry
